# Projections of industrial water withdrawal under shared socioeconomic pathways and climate mitigation scenarios

**DOI:** 10.1007/s11625-016-0392-2

**Published:** 2016-09-13

**Authors:** Shinichiro Fujimori, Naota Hanasaki, Toshihiko Masui

**Affiliations:** 1 Center for Social and Environmental Systems Research, National Institute for Environmental Studies, 16–2 Onogawa, Tsukuba, Ibaraki 305–8506 Japan; 2 Center for Global Environmental Research, National Institute for Environmental Studies, 16–2 Onogawa, Tsukuba, Ibaraki 305–8506 Japan

**Keywords:** Industrial water withdrawal, Shared socioeconomic pathway, Technological assumption, Computable general equilibrium model

## Abstract

We estimated global future industrial water withdrawal (IWW) by considering socioeconomic driving forces, climate mitigation, and technological improvements, and by using the output of the Asia–Pacific Integrated Model/Computable General Equilibrium (AIM/CGE) model. We carried out this estimation in three steps. First, we developed a sector- and region-specific regression model for IWW. The model utilized and analyzed cross-country panel data using historical statistics of IWW for 10 sectors and 42 countries. Second, we estimated historical IWW by applying a regression model. Third, we projected future IWW from the output of AIM/CGE. For future projections, we considered and included multiple socioeconomic assumptions, namely different shared socioeconomic pathways (SSPs) with and without climate mitigation policy. In all of the baseline scenarios, IWW was projected to increase throughout the twenty-first century, but growth through the latter half of the century is likely to be modest mainly due to the effects of decreased water use intensity. The projections for global total IWW ranged from 461 to 1,560 km^3^/year in 2050 and from 196 to 1,463 km^3^/year in 2100. The effects of climate mitigation on IWW were both negative and positive, depending on the SSPs. We attributed differences among scenarios to the balance between the choices of carbon capture and storage (CCS) and renewable energy. A smaller share of CCS was accompanied by a larger share of non-thermal renewable energy, which requires a smaller amount of water withdrawal per unit of energy production. Renewable energy is, therefore, less water intensive than thermal power with CCS with regard to decarbonizing the power system.

## Introduction

Global water withdrawal has been projected to increase dramatically as a result of population and economic growth throughout the twenty-first century (Hayashi et al. 2012; Shen et al. 2008; Hagemann et al. 2013; Hanasaki et al. 2013b; Alcamo et al. 2007; Oki et al. 2003). Moreover, future climate change is projected to alter patterns of precipitation and the hydrological cycle globally, which could further limit available water resources (Nohara et al. 2006). In combination, these changes will cause severe discrepancies between water supply and demand in multiple regions around the world (Hanasaki et al. 2013b).

Industrial production is a major source of water use globally. There are two approaches to estimate future projections of global industrial water withdrawal (IWW). One is to develop statistical regression models of total IWW by nation or region. Alcamo et al. (2007) and Shen et al. (2008) developed a series of regression models to estimate the total IWW of individual countries for which data were available. Alcamo et al. (2007) showed that the historical growth in national IWW is primarily correlated with electricity production. Temporal variations in water use intensity (i.e., IWW per unit of electricity produced) were attributed to structural and technical changes—expressed as a hyperbolic function of gross domestic product (GDP) per capita and a constant rate of annual improvement, respectively. Shen et al. (2008) and Hanasaki et al. (2013a) adopted a similar approach using several economic indicators for which future projections are available. IWW can be subdivided into manufacturing processes and cooling water in power generation. Because the way in which water is used differs substantially between these two processes, efforts were made to make projections separately. Vassolo and Döll (2005) developed a global database of manufacturing and cooling thermal power stations. Furthermore, Flörke et al. (2013) developed a model to project national manufacturing and cooling water usage. Another approach is to develop a regression model of IWW using individual industrial sector data. This is accomplished using sector-specific output and economic models. Hayashi et al. (2012) developed a model that explains IWW by physical production volume and water use efficiency. Kyle et al. (2013) and Davies et al. (2013) assessed the electricity sector and showed how electricity water demand is affected by climate mitigation and technological change. Hejazi et al. (2014a) focused on the bioenergy sector and found that future bioenergy expansion caused by climate mitigation could drastically change water usage globally. Bijl et al. (2016) developed a detailed technological model to project future water use, which took into account improvements in efficiency in both water end-uses and driving forces. Fricko et al. (2016) assessed global energy sector water use and thermal water pollution across a broad range of energy system transformation pathways to assess the water use impacts of a 2 °C climate policy.

From an economic point of view, water is one of the production factors. To project water demand, it is essential to understand the equilibrium of supply and demand of water. Computable general equilibrium (CGE) models are powerful tools (Harou et al. 2009) for evaluating the consequences of water demand due to taxation (altering the supply curve), economic shocks (changes in price and quantity for certain sectors), and other factors (Harou et al. 2009). CGE models have been widely applied to regional and global water resources studies, with a primary focus on the agriculture sector. For example, Diao and Roe (2003) analyzed Morocco’s agricultural trade policies and water distribution trends using a CGE model, and revealed that elimination of agricultural tariffs would shrink domestic agricultural markets, whereas creating water resource markets would contribute to an optimal distribution of water, and would also compensate farmers for any losses due to free trade agreements. van Heerden et al. (2008) analyzed the relationship between income distribution and water use taxes in South Africa using a CGE model. Hassan and Thurlow (2011) assessed water management and distribution in South Africa. Studies have also been conducted on a global scale. Berrittella et al. (2007) developed the GTAP-W (Global Trade Analysis Project-Water) global CGE model, and analyzed the role of international trade under different water scarcity scenarios. They identified the consequences of changes in major macroeconomic indicators such as GDP and welfare. Calzadilla et al. (2010) enhanced GTAP-W by distinguishing between blue and green water, and analyzed the effects of changes in current water sector trends and policies on welfare. Recently, GTAP-W was further updated, in the form of the GTAP-BIO-W (Global Trade Analysis Project-Biofuel-Water) model, which provides more details on the agricultural water withdrawal associated with basin base information (Liu et al. 2014).

Although a number of CGE water studies have been published pertaining to the agricultural sector, IWW has not explicitly been modeled by CGE models. The main reason for this is limited availability of data. To assess IWW using CGE, sector- and region-specific IWW information must be prepared; these data must also be consistent with social accounting matrices (SAMs), which are the base datasets of CGE models. To the best of the authors’ knowledge, such comprehensive global IWW information is not yet available. This study describes how to project future detailed sectoral IWW. Most of the earlier studies that estimated future IWW used multiple regression with population, GDP, and other explanatory variables. This approach is intuitive, but troublesome in the situation where industrial structure changes drastically. In contrast, our approach sums the sector-wise IWW, which better reflects the change in the dominant industrial sector. Although (Hayashi et al. 2012) reported a similar approach, there are few such studies and this study contributes to develop this method. Furthermore, the incorporation of IWW into the CGE model framework expands the capability of the integrated assessment model community to assess water resources more comprehensively and accurately.

Accordingly, in this study, we developed a model to estimate historical IWW, which will help to accommodate IWW within a CGE. In turn, we inputted those data into the Asia–Pacific Integrated Model/Computable General Equilibrium (AIM/CGE) (Fujimori et al. 2012, 2014c; Hasegawa et al. 2014; Fujimori et al. 2014a; Fujimori et al. 2014b; Ishida et al. 2014; Fujimori et al. 2013) and used this model to project future IWW. The aims of this paper are twofold. One aim was to develop a model to estimate IWW that would be compatible and consistent with the outputs of AIM/CGE; and the other was to quantitatively project future IWW using the model, and to analyze the influence of socioeconomic assumptions and climate mitigation measures on the results. For the socioeconomic assumptions, we adopted the use of shared socioeconomic pathways (SSPs; Moss et al. 2010; O’Neill et al. 2014). Some details of SSPs will be discussed in later sections (“Socioeconomic scenarios”). For climate mitigation measures, we focused on the installation of carbon capture and storage (CCS) and the imposition of a high carbon price; the former increases IWW while the latter decreases IWW. Several studies have treated CCS and its water use. For example, Fricko et al. (2016) projected global water use under a climate change mitigation scenario and concluded that CCS, nuclear, and CSP are the major contributors to increased water usage. National and local-scale studies have been conducted for the US, UK, and Brazil (Clemmer et al. 2013; Cameron et al. 2014; Macknick et al. 2012; Byers et al. 2014; Merschmann et al. 2013). Although the highlights of the individual studies differ, they examined several future scenarios and revealed that CCS tends to increase water use. Our coverage of IWW was framed around the industrial sector; agriculture, including the energy crop production sector, is not included in this study.

## Methods

### Overview

**Fig. 1:**
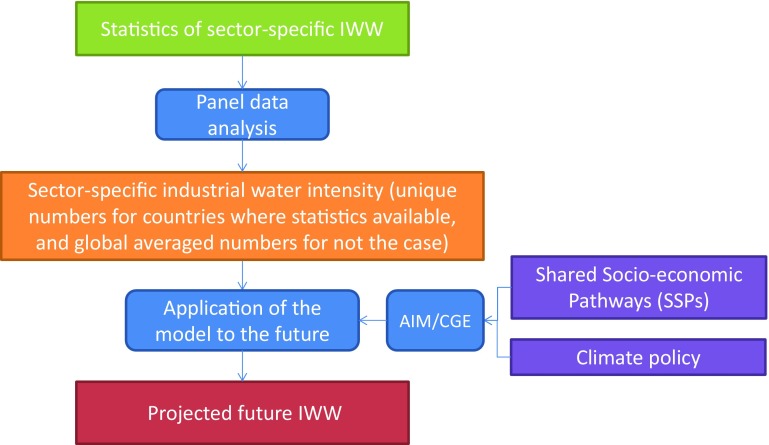
Methodological framework

Figure 1 illustrates the methodological framework used in this study. To develop a model to estimate nation- and sector-specific IWW, we conducted a panel data analysis incorporating information on historic IWW and output indicators. These indicators included energy production, measured in an energy unit (e.g., MWh) in the electricity industry, and the constant price value added, given in monetary units by the other manufacturing industries. We conducted the panel data analysis using cross-country data for each industry, and developed models to estimate IWW for each industry. The 10 industries are “basic metal”, “chemical”, “electricity (excluding hydropower)”, “food processing”, “mining”, “non-metal and mineral”, “paper and pulp”, “textile”, “other manufacturing”, and “industry total” sectors. We then applied this model to historical periods and validated their reproducibility. Finally, we projected future water withdrawal using the outputs of the AIM/CGE model, which were associated with a set of scenarios that incorporated SSPs and climate mitigation policies.

### Panel data analysis for historical water withdrawal

#### Basic concept and formula

The volume of IWW varies considerably among nations. In this study, we dealt with the water use intensity of IWW (IWW per output).

1 $$\gamma_{r,j}^{t} = {{I_{r,j}^{t} } \mathord{\left/ {\vphantom {{I_{r,j}^{t} } {D_{r,j}^{t} }}} \right. \kern-0pt} {D_{r,j}^{t} }},\quad \forall r \in R,\; j \in J,\;t \in T$$

where *I* is IWW, $\gamma_{r,j}^{t}$ is the water use intensity, *D* is the output, and subscripts *r, j, t* denote regions, sectors, and years, respectively. *D* represents the constant price value added for a specific industry in monetary units. Notably, electricity production generated by thermal plants (Notably, electricity production generated by thermal plants (fossil fired, biomass, and nuclear) is measured in MWh in the electricity industry.) Hanasaki et al. (2013a) used Eq. (1) and examined historical changes in $\gamma_{r,j}^{t}$ for 16 countries in great detail. They inputted total IWW for $\gamma_{r,j}^{t}$ and total electricity production for *D* and found that $\gamma_{r,j}^{t}$ decreased over time for all nations, except for a few cases where there was technological improvement and structural change within industries. They also found that $\gamma_{r,j}^{t}$ varied considerably among nations.

In the present study, a panel data analysis was conducted for 10 sectors individually to estimate historic water use intensity in specific countries. We assumed that changes in water use intensity are primarily explained by time, due to the year-by-year technological improvements that we observed.

2 $$\ln \gamma_{r,j}^{t} { = }\, \alpha_{j} \overline{{Y^{t} }} + \beta_{r,j} + \varepsilon_{r,j}^{t}, \quad \forall r \in R,\;j \in J,\;t \in T,$$

where $\alpha_{j}$ is the sector-specific parameter for technological improvement in sector *j*; $\overline{{Y_{t} }}$ is time; $\beta_{r,j}$ is a country-specific water use intensity parameter for region *r* and sector *j*; and $\varepsilon_{r,j}^{t}$ is the error term.

$\alpha_{j}$ is a time trend parameter representing the rate of technological improvement, in terms of water withdrawal, for each industry. Equation (1) implies that water use intensity changes constantly over time. The parameter $\beta_{r,j}$ represents country-specific water technology. As shown in Table 6, for all sectors, the model passes an *F* test; therefore, here we assume fixed effects for all countries. Furthermore, the Hausman-type test showed that 8 of 10 sectors (with the exceptions being the “textile” and “other manufacturing” sectors) are more suited for the application of random effects. Therefore, we used a random effect model for these eight sectors. For the estimation of the random effect model, we applied the method of generalized least squares.

Finally, sector-wise IWW ($I_{r,j}^{t*}$) is estimated as follows:

3 $${I_{r,j}^{t}}^* = I_{r,j}^{t} \times \frac{{I_{{r,\hbox{``}{\text{total}}{\hbox{''}}}}^{t} }}{{\sum\limits_{j} {\gamma_{r,j}^{t} D_{r,j}^{t} } }}$$

where $I_{r,j}^{t*}$ is the updated IWW and $I_{{r,{\hbox{``}}{\text{total}}{\hbox{''}}}}^{t}$ is the IWW total. When we apply the model, the summation of the sector-wise estimation does not necessarily agree with the national total IWW. Therefore, we scaled the sector-wise IWW by the estimated “industry total.” This assumption implicitly assumes that the reported national total IWW is more reliable than the summation of the estimated sector-specific one.

#### Data and application

We collected industry-specific national statistical data on IWW for 43 countries (Office for National Statistics (United Kingdom) 2009; USGS 2004; Industrial statistics 2007; Statistics Canada 2010; Report on the State of the Environment In China 2010; SI-STAT database 2011; The Australian Bureau of Statistics 2006; EUROSTAT 2011) (Slovenia is recorded in both the national statistics and EUROSTAT) and the total national IWW for 85 countries reported in the international AQUASTAT statistics (Table 1). The data cover the period from 1971 to 2005 (at most). Where possible, data were collected at the national or European Union level. Some data were available for a limited number of industries and periods (e.g., the UK reports only a single year of data for all sectors). The exception is Japan, where statistical information is available for all years, from 1971 to 2004, and for all sectors.

**Table 1:** Statistics used in the panel data analysis

Source	Year of publication	Publisher	Number of industrial sectors	Years covered	Countries covered
AQUASTAT	2012	FAO^a^	1 (industry only)	1965–2010 (limited data for some countries)	85
Environmental accounts: consumption of water resources by industrial sector	2009	Office for National Statistics (UK)^b^	15	1997	1
Estimated use of water in the US in 2000	2004	USGS (US)^c^	1 (electricity only)	Every 10 years since 1970	1
Industrial statistics	2007	METI (Japan)^d^	10	1950–2004	1
Industrial water use	2010	Statistics Canada^e^	18	2005–2007	1
Report on the state of the environment in China	2010	Ministry of Environmental Protection of the People’s Republic of China^f^	43	2004–2007	1
SI-STAT database	2011	Statistical Office of the Republic of Slovenia^g^	8	1985–2005	1
Water Account Australia	2010, 2006	Australian Bureau of Statistics^h^	15	2001, 2004, 2008	1
Water Statistics (EUROSTAT)	2011	European Commissions^i^	10	1998–2007	37

*FAO* Food and Agriculture Organization of the United Nations, *USGS* US Geological Survey, *METI* Ministry of economy, trade, and industry

^a^FAO (2012), ^b^ Office for National Statistics (United Kingdom) (2009), ^c^ USGS (2004), ^d^ Industrial statistics (2007), ^e^ Statistics Canada (2010), ^f^ Report on the State of the Environment In China (2010), ^g^ SI-STAT database (2011), ^h^ The Australian Bureau of Statistics (2006), ^i^ EUROSTAT (2011)

The output data of each industry, which are needed to convert water withdrawal into water withdrawal intensity, were collected as follows. For the nine manufacturing sectors (i.e., all of the industrial sectors except for the electricity sector, hereafter the manufacturing sector), the sector-specific current price value added was compiled with reference to the STructural ANalysis database (STAN) (OECD 2005), the UNIDO (United Nations Industrial Development Organization) Industrial Statistics Database (INDSTAT2) (UNIDO 2009), Global Trade Analysis Project (GTAP) (Dimaranan 2006), and Organization for Economic Cooperation and Development (OECD) input–output tables (OECD 2010). Current price value added was then converted into historical time series data to give the constant price value added using deflators and the purchasing power parity (PPP) conversion factor (see Fujimori and Matsuoka (2011) for details). For the electricity sector, electricity production data, in physical units (MWh/year), were used as the output of the sector. The physical unit data, pertaining to power production generated by fossil fuel and nuclear power plants, were derived from the data of the International Energy Agency (International Energy Agency 2013a, b).

Since the observational statistics occasionally included unrealistic records, data were excluded in the following cases:

1. If the annual rate of change in water use intensity exceeded +100 % year^−1^ or fell below −50 % year^−1^ between two reported years; and
2. If the water use intensity for the electricity production sector exceeded 10-fold, or fell below 1/10th, of 21,000 gal/MWh (equivalent to 79.5 m^3^/MWh); this was the water use intensity in the US in 2000 (Freedman and Wolfe (2007).

### Estimation of future water withdrawal

#### Model

The AIM/CGE model was used to project the future output of industrial sectors. AIM/CGE is a 1-year-step recursive-type dynamic general equilibrium model that includes 17 regions and 42 industrial classifications (see Table 7 and Table 8 for the lists of regions and industries, respectively). AIM/CGE includes detailed classifications of the energy and agricultural sectors. The details of the model structure and mathematical formulas are described in the AIM/CGE manual (Fujimori et al. 2012).

Region- and industrial sector-specific economic output is calculated by AIM/CGE and used for the future IWW projection. Among the 42 industries, 11 sectors produce electricity, namely coal-fired power, oil-fired power, gas-fired power, nuclear power, hydroelectric power, geothermal power, photovoltaic power, wind power, waste biomass power, other types of renewable energy power generation, and advanced biomass power generation.

**Table 2:** Scenario framework

	SSP1	SSP2	SSP3	SSP4	SSP5
Baseline	SSP1_BaU	SSP2_BaU	SSP3_BaU	SSP4_BaU	SSP5_BaU
Climate mitigation (3.4 W/m^2^ stabilization)	SSP1_34W	SSP2_34W	SSP3_34W	SSP4_34W	SSP5_34W

#### Climate mitigation scenarios

The scenarios adopted for the future projections have two dimensions: one relates to climate mitigation and the other relates to socioeconomic assumptions (Table 2). There are two scenarios for climate mitigation: baseline with no constraint on greenhouse gas (GHG) emissions; and a stabilization value of 3.4 W/m^2^. As Fujimori et al. (2016) and Riahi et al. (2016). the world under the SSP3 scenario cannot achieve the long-term mitigation target of 2.6 W/m^2^. Therefore, 3.4 W/m^2^ was selected because it is an achievable target for all SSPs.

#### Socioeconomic scenarios

Regarding the socioeconomic dimension, we adopted the SSPs concept. SSPs consist of narrative storylines and quantitative information about plausible future world states. SSPs comprise five representative scenarios characterized by two dimensions: socioeconomic challenges for mitigation and adaptation. For example, SSP1 (“sustainability”) is characterized by low-level socioeconomic challenges for both mitigation and adaptation, which implies a relatively optimistic view of future states in the context of climate change. Such a view is reflected in a more highly educated and smaller population, greater economic growth, more advanced energy technology, and various other factors. Conversely, SSP3 (“regional rivalry”) is characterized by high-level mitigation and adaptation challenges. SSP4 (“Inequality”) has strong income inequality and a high adaptation challenge. SSP5 (“Fossil-fueled development”) has the greatest economic growth with large fossil fuel consumption, and SSP2 (“Middle of the road”) falls somewhere in the middle of the other four scenarios described. More detailed descriptions of specific SSPs are presented in O’Neill et al. (2014).

There are four main elements necessary for the projection of future water withdrawal scenarios considering socioeconomic assumptions: the output of individual industrial sectors, electricity production, the composition of energy sources (taking into account the effects of adopting CCS in coal-fueled power plants), and water use technology.

**Fig. 2:**
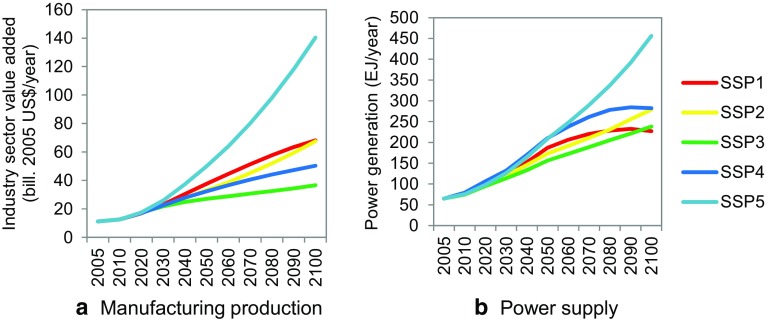
Main driving forces of global industrial water withdrawal (IWW) for the baseline cases

**Fig. 3:**
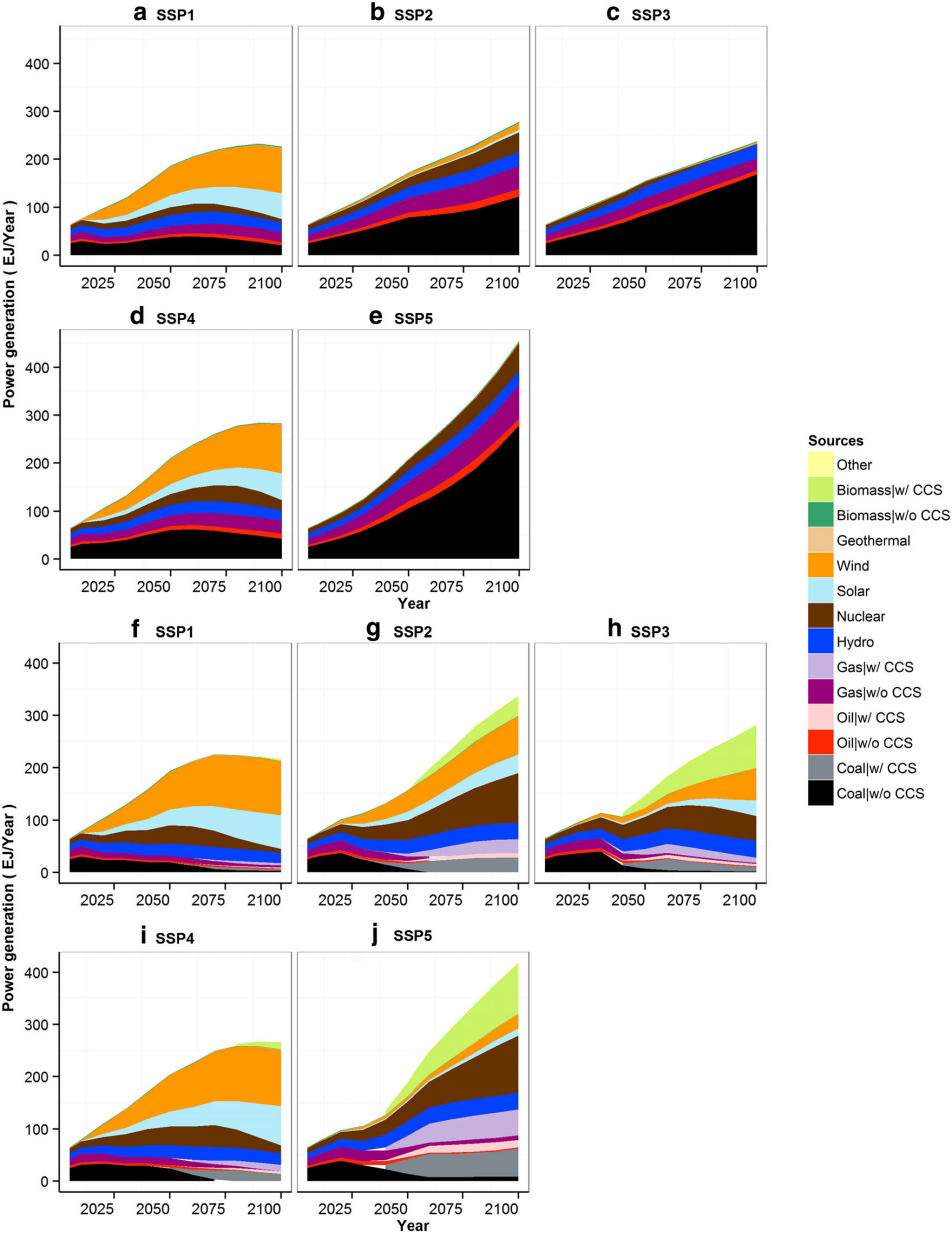
Global electricity energy sources. **a**–**e** are for the baseline and **f**–**j** are for mitigation scenarios

The driving forces (electricity supply and the total manufacturing value added) calculated by AIM/CGE are presented in Fig. 2. These have been prepared for five SSP scenarios for the period 2005–2100. All scenarios project a consistent increase in industrial output throughout the twenty-first century, although the degree of increase varies. For example, SSP5 accompanies the largest amount of electricity production and manufacturing in 2100, as it is characterized by heavy global reliance on fossil fuel consumption and technology. Compared to the other scenarios, SSP4 presents relatively lower industrial output, which is almost stabilized by the latter part of the century. The details of the methods and results can be found elsewhere (Fujimori et al. 2016). Figure 3 provides a brief summary of the composition of energy sources in each SSP, focusing on fossil fuel fired power plants, non-biomass renewable energy (such as wind and solar), and CCS. The types of energy source, as well as the introduction of CCS into the power sector, are also essential for IWW; therefore, we include these factors in Fig. 3 for all 10 scenarios.

Water use technology is assumed to be consistent with the narratives of the SSPs (Table 3). The panel data analysis revealed that industrial sectors have experienced rapid technological progress with respect to water use, whether this high rate of progress will continue or not throughout the century highly depends on socioeconomic conditions. We will discuss this in our Results section, We, thus, assumed that the rate of improvement in intensity would continue throughout the entire twenty-first century in SSP1 and SPP5 (to be consistent with the narratives of these two scenarios pertaining to low-level adaptation challenges). On the contrary, in SSP3 and SSP4, we assumed that challenges for adaptation would be high and the rate of improvement drops to 1/4 of that of SSP1 and SSP5. Finally, we assumed that the rate of improvement is halved in SSP2, which represents a middle course. We discuss the uncertainties associated with these assumptions in “Uncertainty and limitations”.

**Table 3:** Key assumptions regarding the power supply in the different SSPs

	SSP1	SSP2	SSP3	SSP4	SSP5
Consumption of/dependency on fossil fuels	Low	Med	High	High and low*	High
Introduction of non-biomass renewable energy	High	Med	Med	High	Low
Acceptance of CCS	Low	Med	Med	High	Med

*CCS* carbon capture and storage

* Differentiation across income levels and high- and low-income countries is assumed to be high and low, respectively

The minimum achievable water use intensity of the electricity sector, which utilizes water primarily for cooling, is physically constrained. Thus, we assumed 3 m^3^/MWh to be the lowest water use intensity, and that any improvements would stop once regions achieved this level of technology. This intensity level corresponds to the minimum water requirement of closed-loop water reuse technology (EPRI 2002).

The water requirement associated with the introduction of CCS is not captured in the historical data. Therefore, we assumed that CCS doubles the water requirement, in accordance with Kyle et al. (2013).

## Results

### Results of historical panel data analysis

Table 4 shows the estimated $\alpha_{j}$ for all sectors that show a change in water use intensity—mainly due to technological progress—and the associated t statistic for each sector.$\alpha_{j}$ is negative for all sectors, indicating that they all experienced progress in water technology. We took these numbers to equal the annual improvement rate.^1^ In the electricity sector, water use intensity has been decreasing at a rate of 3 % per year, although in total, the water use in this sector has been increasing because electricity production has also been increasing (by as much as 3.1 % per year). $\alpha_{j}$ for the other heavy industries, such as the basic metal, chemical, non-metal and mineral sectors, varies. For example, $\alpha_{j}$ for the basic metal sector is as low as 1.4 %, while the chemical sector shows a $\alpha_{j}$ as high as 4.5 %. Note that original data for these sectors were only available for European countries and Japan. Since the number of records in Japan is larger than in any other country (obtained over a period of 34 years), the overall results may disproportionally reflect historical changes in Japan.

**Table 4:** Results of panel data analysis

	$\alpha_{j}^{{}}$			Intercept			Number of countries	Number of data points
	Estimates	*t* stat		Estimates	*t* stat			
Industry total	−0.011	−2.7	***	−3.900	−21.0	***	77	225
Basic metal	−0.014	−4.8	***	−1.920	−6.6	***	7	60
Chemistry	−0.045	−13.5	***	−1.660	−5.1	***	12	82
Electricity	−0.031	−6.1	***	0.445	1.8	*	8	60
Food processing	−0.021	−9.0	***	−3.595	−23.4	***	11	75
Mining	−0.038	−2.1	**	−1.445	−1.9	*	7	30
Non-metal and mineral	−0.037	−12.8	***	−2.982	−11.5	***	4	38
Paper and pulp	−0.016	−4.5	***	−2.823	−9.7	***	13	91
Textile	−0.034	−9.2	***				11	79
Other manufacturing	−0.089	−4.6	***				7	27

*** *P* < 0.01, ** *P* < 0.05, * *P* < 0.10

The results of a *t* test demonstrated that $\alpha_{j}$ for all sectors differs significantly from zero (the t-value is shown in Table 4). Note that the availability of the data varies among sectors (Table 4).

The estimated historical total IWW for selected countries was compared with the AQUASTAT database, which provides more than three records between 1970 and 2005 (Fig. 8). The generally good agreement between the estimated historical total IWW and AQUASTAT database further supports the validity of the model and methods proposed.

### Future global IWW scenarios overview

**Fig. 4:**
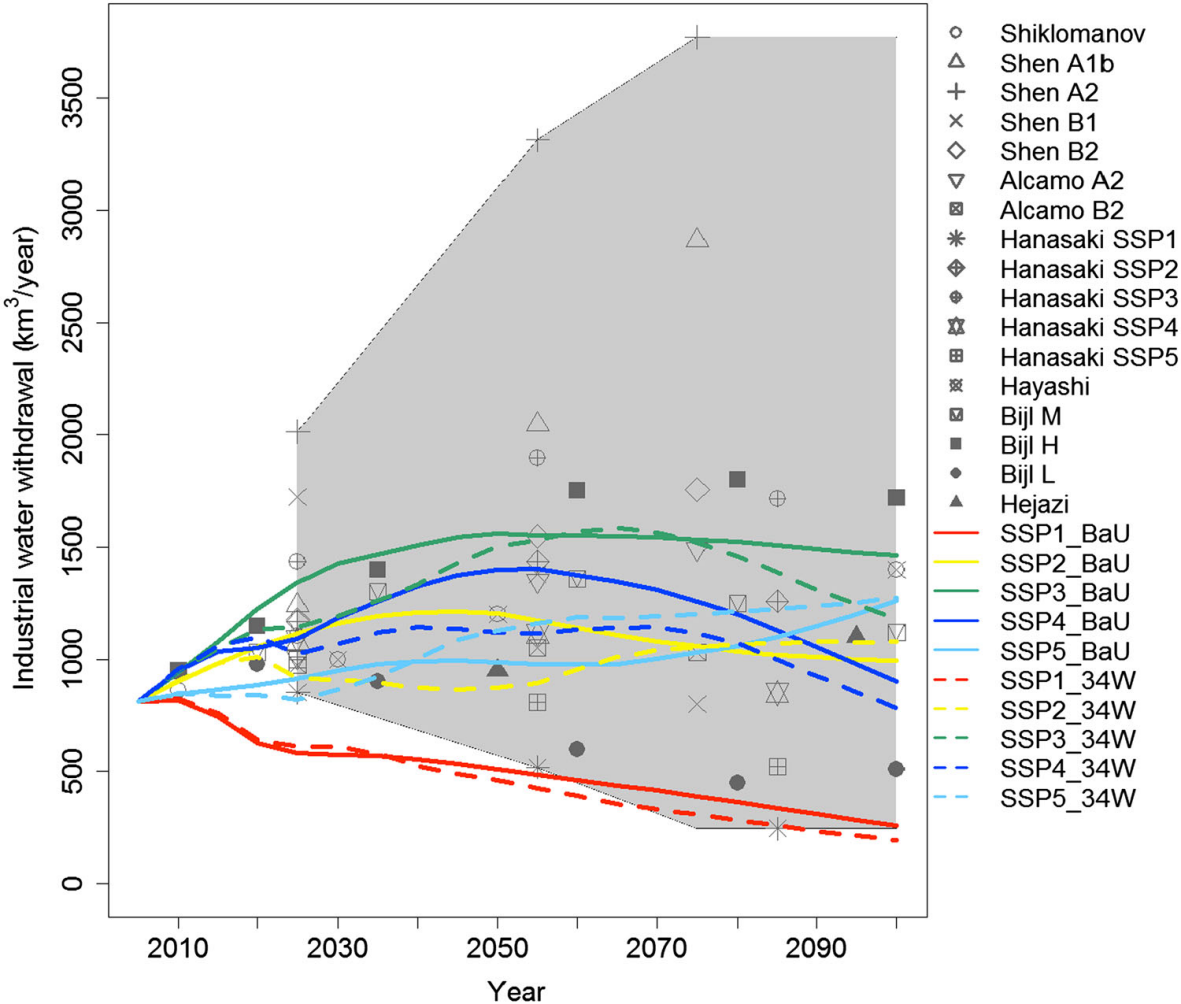
Projected total global IWW compared with the results of existing studies (Shiklomanov 2000; Alcamo et al. 2007; Shen et al. 2008; Hanasaki et al. 2013a; Hayashi et al. 2012; Bijl et al. 2016; Hejazi et al. 2014b)

Figure 4 illustrates 10 future global total IWW scenarios, including projections from earlier studies; a comparison of these projections will be presented later. For the baseline cases (solid lines), withdrawal in 2100 ranged from 257 km^3^/year in SSP1 to 1464 km^3^/year in SSP3. IWW for SSP2 was projected to increase until 2045 and decrease thereafter. This is primarily attributable to the assumptions of continuous improvement in water technology and a modest increase in driving forces. IWW for SSP1 is substantially lower than for SSP2 over the entire period. IWW in SSP5 is lower than in SSP2 for most of the period, except at the end of the century. The rates of growth of GDP and energy consumption in SSP5 are the largest among all the scenarios; technological improvements in water use in SSP5, which are as significant as those in SSP1, suppressed the increase in IWW. As for SSP3, the power generation mix and its total amount looks similar to SSP2 (Fig. 3), but the IWW is higher than in SSP2. This is mainly due to the assumption that technological progress was slower in SSP3 than in SSP2. SSP4 is also assumed to be a world with slow technological progress. Compared with SSP2, the power generation relies to a much greater extent on renewable energies, which consume relatively less water; hence, SSP4 is projected to be lower than SSP2. This is consistent with the narratives of these SSPs, which project high-level adaptation challenges.

IWW for the climate mitigation cases (dashed lines) is generally lower than that for baseline cases. IWW for SSP1 is the lowest among all of the SSPs. Global IWW in 2100 is as low as 196 km^3^/year, which is approximately 40 % of the baseline case. This is mainly due to the assumption in SSP1 of a preference for renewable energies, such as solar and wind power, which require no water for generation. IWW for SSP3 in 2100 is lower than that of the baseline, but reaches as high as 1,181 km^3^/year by 2100. SSP3 needs to reduce GHG emissions by cutting off power generation, since CCS is not available for fossil fuel fired power plants (see Methods section). Carbon prices for SSP3 are notably high (see Fig. 7). Climate mitigation in SSP2, SSP4, and SSP5 is close to that of the baseline cases. This is mainly due to the large uptake of CCS. As explained in the Methods section, CCS is assumed to double water use intensity. Therefore, even though the total amount of fossil fired power generation decreases, the degree of water withdrawal does not. Indeed, IWW in mitigation cases was slightly increased in SSP2 and SSP5.

### Future IWW scenarios by region

**Fig. 5:**
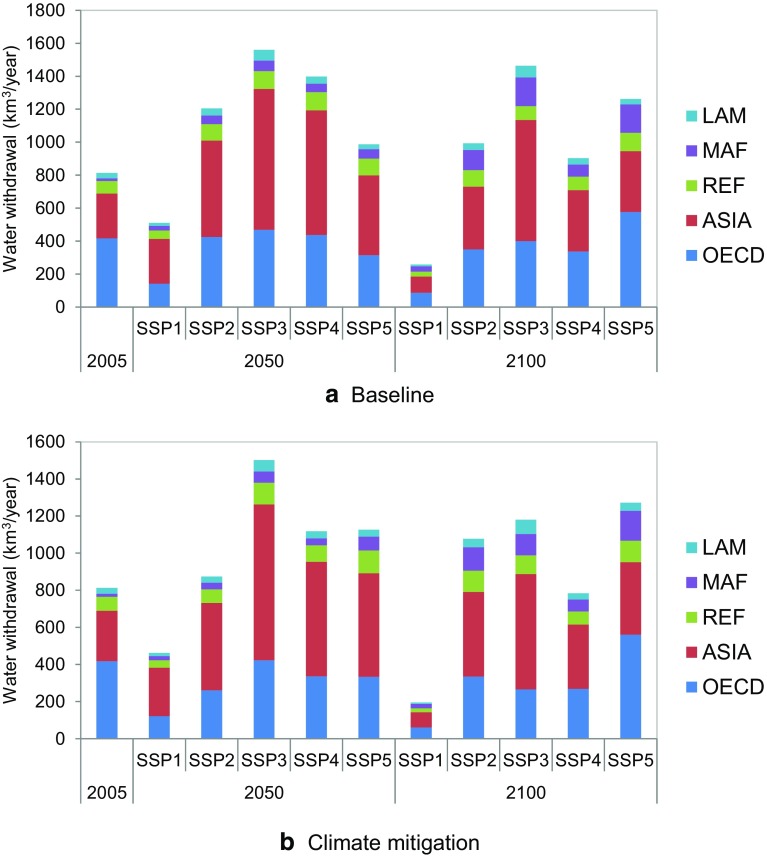
Regional breakdown of projected IWW using shared socioeconomic pathways (SSPs)

The regional breakdowns of future IWW scenarios, in 2005, 2050, and 2100, are shown in Fig. 5 for baseline and climate mitigation cases. The proportion of Asia is largest across scenarios in 2050, but it decreases toward 2100 both in baseline and mitigation scenarios. The proportion of Asia is largest across scenarios in 2050, but it decreases toward 2100 in both the baseline and mitigation scenarios. Asia is expected to have larger GDP growth than the other regions in the first half of this century and that drives IWW (with the main contribution from China). In contrast, in the second half of this century, such economic expansion is stabilized and the water use intensity improvement factor becomes the major factor for projecting IWW. IWW in the Middle East and Africa (MAF) continuously increases: the fraction of MAF IWW in 2100 is larger than that in 2050 in all scenarios, but particularly for SSP1, SSP2, and SSP5, where it accounts for as much as 26, 21, and 21 % of total IWW, respectively. The OECD is projected to maintain a relatively large share of global IWW throughout the twenty-first century. The highest share is seen in 2100 for SSP5, a scenario characterized by fossil fuel development. Among the SSPs, SSP5 has the largest economic growth in high-income countries. The effect of mitigation on the regional distribution of IWW is marginal under this scenario. This is likely because the carbon price is assumed to be consistent globally; hence, all regions face drastic and consistent power system changes.

Figure 5 Regional breakdown of projected IWW by Shared Socio-economic Pathways using shared socioeconomic pathways (SSPs)

### Future IWW scenarios by sector

**Fig. 6:**
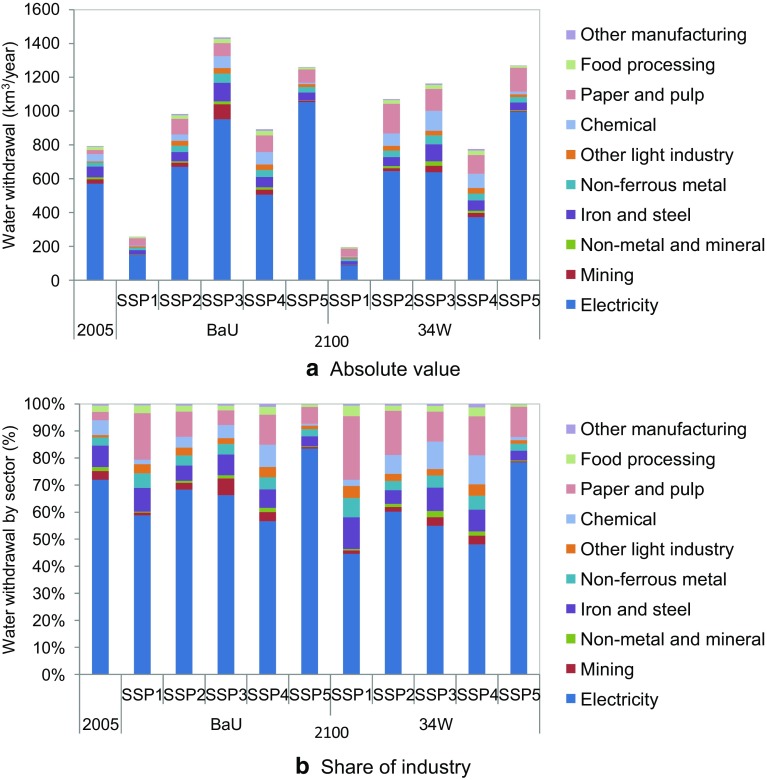
IWW by sector in 2005 and projected withdrawal in 2100. The sectoral classification is AIM/CGE shown in Table 8

**Fig. 7:**
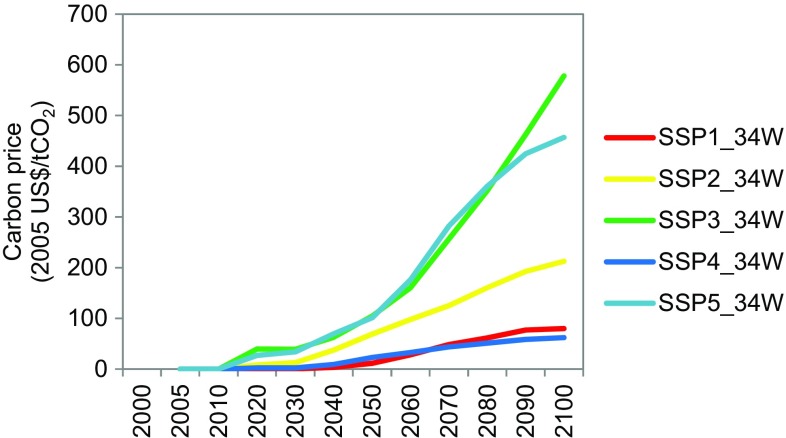
Carbon prices for mitigation scenarios

**Fig. 8:**
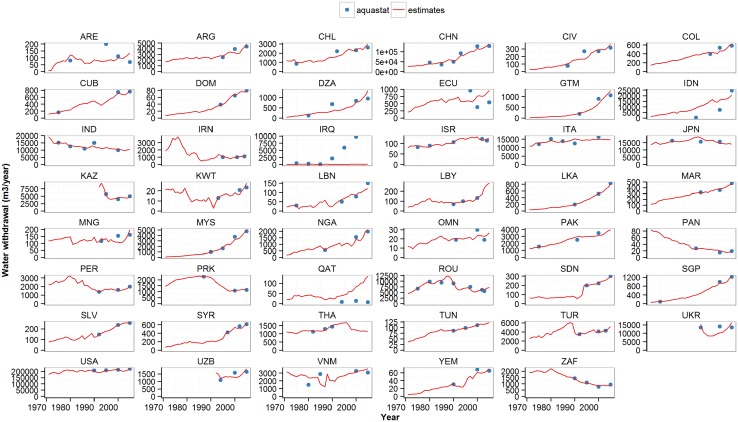
Historical estimates of national IWW and reported values in AQUASTAT

Figure 6 shows the absolute volume and percentage shares of global IWW by sector for 2005 and 2100, for the baseline and mitigation cases. The percentage share of the electricity sector in 2100 for SSP1 and SSP5 baselines is relatively higher than that of the other scenarios, while SSP2, SSP3, and SSP4 have almost the same electricity share as in 2005. There are several factors in SSP1 and SSP5 that may have increased the electricity share. First, strong water use intensity improvement is assumed. Second, electricity demand with relation to GDP is relatively higher than in the other scenarios. Third, total industrial production in relation to GDP is relatively lower than in other scenarios. These factors are based on the SSP narratives (Table 3).

When we look at the mitigation scenarios, sector-specific water withdrawal is notably different from baseline measures. First, the fraction attributed to the electricity sector decreases from 2005 onwards. This is mainly due to two factors: the increase in IWW in sectors other than the power sector due to the introduction of CCS; and the decrease in IWW in the power sector due to the use of renewable energy. Although CCS for thermal power may increase IWW, the effects of renewable energy compensate for this trend. SSP1 is a typical case: installation of CCS is limited and the world relies heavily on renewable energy in the power sector (Fig. 3). There was also a significantly reduced IWW in the electricity sector (from 715 to 213 km^3^/year in 2100). SSP3 also showed a decrease in renewable energy (1266–663 km^3^/year in 2100). By contrast, the differences are relatively small in SSP2, SSP3, and SSP4, because of the restricted availability of CCS in these scenarios (see Table 4).

## Discussion

In this section, the estimated IWW is compared with that of independent reports (Sects. “Estimated historical industrial water withdrawal and comparison with past estimates” and “Comparison of future IWW values with existing estimates”). Sections “Implications for future IWW” and “Uncertainty and limitations” discuss the implications and limitations of our estimations

### Estimated historical industrial water withdrawal and comparison with past estimates

As mentioned earlier, Vassolo and Döll (2005) reported the first global estimates of IWW, which they subdivided into manufacturing water and cooling water. We compared the estimated historical manufacturing and cooling water data (by continent) to the results of Vassolo and Döll (2005), and the results are shown in Table 5.

**Table 5:** Comparison of the results of the present study with those of Vassolo and Döll (2005)

	Cooling of thermal power stations (km^3^/year)			Manufacturing (km^3^/year)**		
	This study		Vassolo and Döll (2005)	This study		Vassolo and Döll (2005)
Year	2005	1995	1995	2005	1995	1995
North America	219.19	195.38	224.40	35.19	40.79	42.53
Latin America	14.93	8.54	7.31	18.40	17.52	21.39
Africa	7.78	4.66	3.64	5.52	3.88	6.22
Europe	176.06	149.57	121.79	50.02	49.70	96.59
West Asia	2.70	1.44	1.46	0.55	0.42	2.72
Asia	147.40	87.95	41.03	150.81	78.59	149.42
Oceania	2.85	2.07	1.14	0.46	0.63	5.93
World	570.92	449.61	400.77	260.96	191.54	324.79

** Our study includes mining while that of Vassolo and Döll (2005) does not

The manufacturing and cooling water cooling values in the two studies agreed well for the North American data, for which the largest volume of IWW is reported. For example, cooling water was estimated at 195 and 219 km^3^/year in 1995 and 2005, compared to 224 km^3^/year in Vassolo and Döll (2005). The present study estimated that cooling water in Europe reached ~149 km^3^/year in 1995, which is higher than the estimate of Vassolo and Döll (2005) (122 km^3^/year). Conversely, our estimate of manufacturing water in Europe was 50 km^3^/year in 1995 and 2005, which is substantially lower than the estimate of 96 km^3^/year reported by Vassolo and Döll (2005). The difference may be attributed to the estimates for the former Soviet Union and other Eastern European countries. Our estimates for Europe rely on the EUROSTAT database, which mainly covers Western European countries. Our estimates for the former Soviet Union and other Eastern European countries were derived from a regression model based on a worldwide regression analysis (see “Panel data analysis for historical water withdrawal”). For example, cooling water in the former Soviet Union was estimated to be as high as ~60 km^3^/year, which is likely to have contributed to the difference in results.

Results for Asia differed greatly between the two studies. We estimated that China, India, and Japan used the most water, accounting for 47, 13, and 10 km^3^/year of thermal power withdrawal, respectively. The estimated volume of cooling water was ~85 km^3^/year in 1995, more than twice the value of 41 km^3^/year reported by Vassolo and Döll (2005). The Chinese estimate helps to explain this difference. First, the discrepancies are primarily attributed to the differences in the statistics used in the two studies. The statistics used for China in the present study are official Chinese data (Report on the State of the Environment In China 2010). The records started in 2004 (42 km^3^/year for thermal power). The statistics used in Vassolo and Döll (2005) were taken from Carmichael and Strzepek (1987) and only the steel sector data are available from this study (Carmichael and Strzepek (1987). The worldwide average water use intensity statistics for other sectors were applied in China. Meanwhile, the present study used Chinese statistics for individual sectors (as well as Japanese statistics). Our results would be more reliable if more recent, national statistics provided more reliable information.

Oceania accounts for a much smaller proportion of global total water use, but estimates from our study, and that of Vassolo and Döll (2005), differ considerably, particularly for the manufacturing sector. Considering the scale of its economy and industrial sector, Australia dominates water consumption in Oceania. The present study estimated a withdrawal value of 0.63 km^3^/year for manufacturing, whereas Vassolo and Döll (2005) reported a value of 5.93 km^3^/year. The estimate of Vassolo and Döll (2005) is inconsistent with the values generated by The Australian Bureau of Statistics (2006), which reported manufacturing and mining water withdrawal values of 0.21 and 0.544 km^3^/year, respectively, in 1996. The Australian Bureau of Statistics (2006) reports on water consumption in the electricity sector, but we could not use those data because they included hydropower; we excluded hydropower-related consumption data since they are hard to define.

As previously detailed, estimates of IWW differ between the present study and that of Vassolo and Döll (2005). Estimates for sector-specific IWW in our study were based on national statistics if it is available; for countries lacking national statistics, estimates were based on AQUASTAT data for total withdrawal, and water use intensity was estimated from sector-specific water withdrawal data. While Vassolo and Döll (2005) based their calculations on industrial production and water use intensity data derived from literature, this does not necessarily guarantee consistency with sector-specific IWW reports. Differences in the statistics used seem to be a major factor underlying differences in the estimates between the studies. However, evaluating the degree to which estimates are realistic is difficult because we cannot evaluate the reliability of the statistics. The methodology used in this study has two advantages: first, it constrains sector-specific IWW to nationally reported values where available; and second, it has the potential to incorporate updated information about water withdrawal for a given sector, assuming that national statistics are more reliable than other widely used statistics, such as those provided in the AQUASTAT database.

### Comparison of future IWW values with existing estimates

Figure 4 illustrates our 10 scenarios, along with estimates from previous studies. A1, A2, B1, and B2 denote SRES (Special Report on Emissions Scenarios). When compared with other projections, our estimates are mostly within the range of the previous studies but tend to be lower, particularly in the late twenty-first century. Note that the upper edge of earlier projections is formed by the plots of Shen et al. (2008). Hence, our projections are close to earlier estimates, except for those put forward by Shen et al. (2008). Notably, our projection for SSP1 with climate mitigation (SSP1_34 W) is out of range in the first half of the study period. Since none of the earlier studies plotted here take into account climate mitigation, it might not be appropriate to compare the climate mitigation case directly with earlier studies.

Regarding sector-specific future estimates, Davies et al. (2013) and Kyle et al. (2013) projected electricity water withdrawal for electricity generation using the Global Change Assessment Model (GCAM); our projection trends agree with theirs. When comparing the baseline simulations, our estimates (SSP2 baseline) for 2100 are slightly higher: IWW for the electricity sector in the present study’s SSP2 for 2100 is 806 km^3^/year, while that of Davies et al. (2013) is around 550 km^3^/year by 2095.^2^ This could be due to differences in the electricity demand.

Kyle et al. (2013) projected water withdrawal for electricity generation under various climate mitigation assumptions, based on Davies et al. (2013), and showed that global total volume was around 300–550 km^3^/year in 2100. The range is due to differences in the technology adopted to produce power, such as nuclear, fossil fuel with CCS, and renewables. They concluded that climate mitigation decreases, or does not change, water withdrawal for electricity generation, which is similar to our estimates (Fig. 6).

### Implications for future IWW

From the results shown in “Future global IWW scenarios overview”, several implications can be drawn. First, the projections of IWW are sensitive to socioeconomic assumptions, particularly for energy sources tied to electricity generation. Indeed, we have shown that IWW depends on technical aspects in power generation sectors, such as the availability of CCS and the development of renewable energy. Earlier studies, such as that of Hanasaki et al. (2013b), assessed the impact of climate mitigation policy on water scarcity, but did not account for a technological shift accompanying mitigation policies, which underestimates the influence on IWW. We have demonstrated that climate mitigation would potentially have both positive and negative effects on IWW. Note that this study does not include potential water needs for producing bioenergy crops. Hejazi et al. (2014a) implied that a considerable volume of irrigation water would be needed to implement stringent climate mitigation policies, which in turn implies that climate policy may further impact total water demand.

Second, from the results shown in “Future IWW scenarios by sector”, it can be seen that sectoral differences among the scenarios are relatively small. The electricity sector is projected to be the largest IWW sector. However, some scenarios, such as SSP3 with climate mitigation, showed an increase in the non-electricity sector, which came to account for almost half of the total IWW. Therefore, there should be further investigation of this issue in non-electricity sectors, especially in scenarios where CCS availability is limited.

The other implication of this study is that future IWW is highly dependent on assumptions pertaining to water use technology. SSP1 has a higher GDP, and a higher proportion of electricity generated by fossil fuel fired power plants, than SSP2, but IWW in SSP1 is smaller than in SSP2; this is mainly due to its particular water use technology assumption. Future technology is hard to predict and we are facing large uncertainties when deriving these estimates. This also indicates that we should insure ourselves against such uncertain situations. This study provides an additional perspective on industrial water use for SSP scenarios, and enhances our understanding of these scenarios.

### Uncertainty and limitations

We identified several sources of uncertainty, as well as several limitations. First, future projections rely on assumptions regarding technological improvements. Technological progress associated with time was statistically significant in the panel data model analysis of historical data, but it is highly uncertain whether these results can be extrapolated indefinitely into the future. If additional statistical information becomes available, other types of regression function could be validated and different scenarios could be created. Thus, a further set of statistical data is needed that can provide more realistic estimations, as well as decrease the magnitude of uncertainty in the estimates. Second, the panel data analysis was based mainly on data from developed countries, with the results then extrapolated to developing countries, which may have introduced bias. This limitation will also be overcome as more statistical data from developing countries becomes available. Third, we aggregated overall water technology into a single factor of water use intensity, a technique that may have overlooked many individual technological factors. For example, water reuse technology is an important and practical means of reducing water consumption, but we were unable to incorporate it explicitly because insufficient data were available. Additionally, the assumptions used for future technological improvements were based on historical estimates, but those used for SSPs were selected arbitrarily.

### Conclusions

The objectives of this paper were two-fold. First, we aimed to add to current global IWW knowledge by means of detailed sectoral classifications that are consistent with the AIM/CGE industrial classification. Second, we aimed to project IWW using an AIM/CGE model output. Moreover, for the latter objective, we assessed how socioeconomic conditions and climate policy affects the IWW. For the first objective, we applied a panel data analysis to cross-sectional data from 42 countries to estimate historical IWW, focusing on sector-specific technological improvements. For the second objective, based on the above estimates, we projected plausible future scenarios, which were coupled with the narratives of the SSP scenarios and climate mitigation policies, by using AIM/CGE. In all of the SSP baseline scenarios, IWW was projected to increase consistently throughout the twenty-first century, but the latter half of the century tended to show a modest increase or even a slight decrease. The effect of climate mitigation on IWW was in either a negative or positive direction, depending on the SSPs. For example, SSP1 and SSP3 decrease from baseline, while other SSPs are close to the baseline cases. This is mainly due to CCS availability and the supply of renewable energy. If CCS is not preferable, power systems may opt for renewable energy sources instead of fossil fuel fired power plants, which decreases water withdrawal. On the other hand, SSP2, SSP4, and SSP5 show few differences between baseline and mitigation cases.

This paper developed IWW data by coupling it with a CGE model. This will enable us to conduct further analyses incorporating climate mitigation in conjunction with climate change impact studies. The application of SSPs to water assessment research represents another contribution to the scientific community. SSPs provide a community-based platform for the interdisciplinary assessment of climate change impacts. This study demonstrates how SSPs can be interpreted for water use assessment.

Further studies are needed to improve the methodology for making future IWW projections, particularly by taking into account the physical parameters of specific industrial sectors. For example, the production of iron and steel is described in terms of a physical unit in steel sector statistics (e.g., (Hayashi et al. 2012), thereby enabling more precise analysis. The models developed in this study should be incorporated into the CGE modeling framework to analyze, for example, the price adjustment effect. The estimates generated in this study could be used in conjunction with the SAM, a database for the CGE model, which may have potential in terms of integrated assessments of climate mitigation and adaptation.

## Appendix

General description of AIM/CGE

AIM/CGE is a computable general equilibrium model that covers all economic goods and considers interactions among production factors. In the model, supply, demand, investment, and trade are described in terms of individual behavioral functions that respond to changes in the price of production factors and commodities, as well as to changes in technology and preference parameters. Production functions are formulated as multi-nested constant elasticity substitution (CES) functions. Household demand functions are formulated as linear expenditure system (LES) functions. For trade, substitution between domestic and imported commodities is based on the Armington assumption, and a CES function is used for aggregation of domestic and imported commodities. Disaggregation between exports and domestic supply is described by a constant elasticity transformation (CET) function. A single international trade market is assumed for each traded commodity. Allocation of land by sector is formulated as a multi-nominal logit function to reflect differences in substitutability across land categories with land rent. In a standard setup, AIM/CGE deals with 17 regions (Table 6) and 42 industries (Table 8).

See Tables 6, 7, 8 and Figs. 7, 8.

**Table 6:** Panel data analysis: *F* test and Hausman-type test

	*p* value for *F* test	*p* value for Hausman-type test
Industry total	5.82E−55	0.94
Basic metal	2.47E−29	0.79
Chemistry	6.51E−44	0.89
Electricity	5.86E−28	0.65
Food processing	1.79E−26	0.82
Mining	5.90E−20	0.85
Non-metal and mineral	4.94E−09	0.18
Paper and pulp	2.97E−42	0.80
Textile	1.42E−24	0.01
Other manufacturing	1.82E−07	0.03

**Table 7:** Region codes

Code	Region	Code	Region
JPN	Japan	TUR	Turkey
CHN	China	CAN	Canada
IND	India	USA	United States
XSE	Southeast Asia	BRA	Brazil
XSA	Rest of Asia	XLM	Rest of South America
XOC	Oceania	XME	Middle East
XE25	EU25	XNF	North Africa
XER	Rest of Europe	XAF	Rest of Africa
CIS	Former Soviet Union

**Table 8:** Industrial classifications of AIM/CGE

Agricultural sector	Energy supply sector	Other production sectors
Rice	Coal mining	Mineral mining and other quarrying
Wheat	Oil mining	Food products
Other grains	Gas mining	Textiles, apparel, and leather products
Oil seed crops	Petroleum refinery	Wood products
Sugar crops	Coal transformation	Paper, paper products, and pulp
Other crops	Biomass transformation (1st generation)	Chemical, plastic, and rubber products
Ruminant livestock	Biomass transformation (2nd generation with energy crop)	Iron and steel
Raw milk	Biomass transformation (2nd generation with residue)	Nonferrous products
Other livestock and fisheries	Gas distribution	Other manufacturing
Forestry	Coal-fired power	Construction
	Oil-fired power	Transport and communications
	Gas-fired power	Other service sectors
	Nuclear power	CCS service
	Hydroelectric power	
	Geothermal power	
	Photovoltaic power	
	Wind power	
	Waste biomass power	
	Other renewable energy power generation	
	Advanced biomass power generation	

*CCS* carbon capture and storage

## Footnotes

1 In precise terms, the annual improvement rate is derived from (1-exp($\alpha_{j}$)). However, the obtained values are almost the same as those that were directly estimated $\alpha_{j}$.

2 What Davies et al. (2013) call a median case, in which they assume median technology water use intensity for the base year estimates.
